# Low Genetic Differentiation across Three Major Ocean Populations of the Whale Shark, *Rhincodon typus*

**DOI:** 10.1371/journal.pone.0004988

**Published:** 2009-04-07

**Authors:** Jennifer V. Schmidt, Claudia L. Schmidt, Fusun Ozer, Robin E. Ernst, Kevin A. Feldheim, Mary V. Ashley, Marie Levine

**Affiliations:** 1 The Department of Biological Sciences, University of Illinois at Chicago, Chicago, Illinois, United States of America; 2 The Shark Research Institute, Princeton, New Jersey, United States of America; 3 The Pritzker Laboratory for Molecular Systematics and Evolution, The Field Museum, Chicago, Illinois, United States of America; American Museum of Natural History, United States of America

## Abstract

**Background:**

Whale sharks are a declining species for which little biological data is available. While these animals are protected in many parts of their range, they are fished legally and illegally in some countries. Baseline biological and ecological data are needed to allow the formulation of an effective conservation plan for whale sharks. It is not known, for example, whether the whale shark is represented by a single worldwide panmictic population or by numerous, reproductively isolated populations. Genetic analysis of population structure is one essential component of the baseline data required for whale shark conservation.

**Methodology/Principal Findings:**

We have identified 8 polymorphic microsatellites in the whale shark and used these markers to assess genetic variation and population structure in a panel of whale sharks covering a broad geographic region. This is the first record of microsatellite loci in the whale shark, which displayed an average of 9 alleles per locus and mean H_o_ = 0.66 and H_e_ = 0.69. All but one of the eight loci meet the expectations of Hardy-Weinberg equilibrium. Analysis of these loci in whale sharks representing three major portions of their range, the Pacific (P), Caribbean (C), and Indian (I) Oceans, determined that there is little population differentiation between animals sampled in different geographic regions, indicating historical gene flow between populations. F_ST_ values for inter-ocean comparisons were low (P×C = 0.0387, C×I = 0.0296 and P×I = −0.0022), and only C×I approached statistical significance (p = 0.0495).

**Conclusions/Significance:**

We have shown only low levels of genetic differentiation between geographically distinct whale shark populations. Existing satellite tracking data have revealed both regional and long-range migration of whale sharks throughout their range, which supports the finding of gene flow between populations. Whale sharks traverse geographic and political boundaries during their life history and interbreed with animals from distant populations; conservation efforts must therefore target international protection for this species.

## Introduction

The whale shark, *Rhincodon typus* (Smith, 1828), is the largest shark and the largest fish [1] (for general reviews of whale shark biology see [2], [3]). They reach lengths of 18 meters or more, and can weigh 20 tons [4]. A single female shark caught with more than 300 live embryos in her uteri demonstrated that the whale shark is ovoviviparous [5]. Whale sharks are believed to reach sexual maturity when they are 8–9 meters in length, as most males less than 8 meters have claspers that are not yet fully developed [2]. Estimates of whale shark growth rates suggest that animals 8 meters in length are likely 25–30 years of age [6]. Whale sharks are found in tropical and warm temperate waters around the globe. An epipelagic oceanic and coastal species, they are filter feeders, and have been observed feeding on copepods in Bahia de Los Angeles, coral spawn in Western Australia, and snapper spawn in Belize [7]–[9]. While whale sharks are largely solitary animals, groups of 100 or more are found in seasonal aggregations often associated with spawning events. The whale shark is known to be a highly migratory species, though the frequency and distance of these migrations is the subject of some debate. While occasional trans-oceanic migrations have been reported, most satellite tracking studies show sharks moving within their oceanic region [10]–[15].

Whale sharks have been the target of widespread active fisheries in the past, and while they are currently protected in many waters, open fisheries remain in several countries. Whale sharks are listed as Vulnerable in the IUCN Red List of Threatened Species, and in 2002 the species was placed on CITES Appendix II. A slow growth rate and late time to sexual maturity make animals such as the whale shark particularly slow to recover from overfishing or habitat disruption, and current evidence indicates that whale sharks are declining in number. Aerial surveys, mark-recapture and photo identification have been used to track the abundance over time of whale sharks in various regions. While not all studies agree, most data suggest that whale shark aggregations have fewer sharks of smaller average size in recent years [12], [16]–[18]. As these smaller animals are more likely to be sexually immature, such studies suggest that larger animals of reproductive age have been selectively removed to supply the active market for whale shark flesh and fins [14], [19], [20]. These juvenile feeding aggregations cannot be breeding populations, so it remains unknown where and how the whale shark breeds, and to what extent breeding crosses geographic boundaries. Such questions are not only of biological interest, but are of key conservation importance as well.

The use of microsatellites as a tool to understand the population genetics of a species has revolutionized the field of conservation biology [21], [22]. These repetitive sequences undergo mutations that add or subtract repeat units, and they are therefore highly polymorphic. They provide excellent resolution for assessing intraspecific genetic variability and differentiation. Here we employ microsatellite analysis to evaluate levels of genetic variability across a global panel of whale sharks, and to determine whether sharks from different regions comprise geographically restricted breeding populations. This manuscript reports the first identification and analysis of whale shark microsatellites. These analyses demonstrated moderate levels of genetic diversity within the species as a whole, but little evidence for population structure between different geographic regions.

## Methods

### Collection of whale shark tissue samples

**Figure 1:**
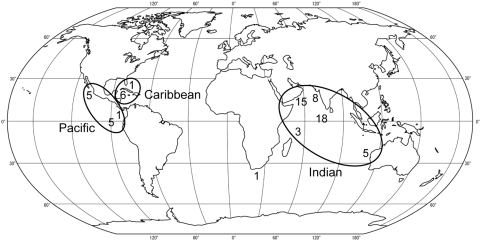
Map of whale shark sample collection sites. The numbers indicate the number of individuals analyzed from each location. The circles indicate the samples pooled into three ocean-based groups for some data analysis—Pacific, Caribbean, and Indian. The one animal from South Africa was included in the Indian Ocean population for all analyses conducted on ocean-specific groupings.

A total of 68 whale shark samples were collected from 11 different sites: Veraval, India (8); Utila, Bay Islands, Honduras (6); Galapagos Islands, Ecuador (5); Dania Beach Florida, USA (1); Mossel Bay, South Africa (1); Mahe, Seychelles (3); Cocos Island, Costa Rica (1); Ningaloo Reef, Western Australia (5); Gulf of Tadjoura, Djibouti (15); Bahia de La Paz, Baja California, Mexico (5); and Male, Maldives (18) (Figure 1). These samples represent the majority of the geographic range of the whale shark, though the study does not encompass animals from the western Pacific Ocean. Tissue samples were harvested by biopsy dart, or retrieved from fishery specimens over the period 2001–2007, and all necessary national and local permits were obtained. Although this sample set is relatively small in number when compared to population genetics studies of abundant species, whale sharks are particularly difficult to locate and sample; the samples analyzed here represent 7 years of effort by many highly competent field biologists. Samples were stored in DMSO tissue buffer (20% DMSO, 0.25 M EDTA, pH 8.0, saturated NaCl) until DNA extraction. Genomic DNA was extracted from whale shark samples by proteinase K digestion, followed by phenol∶chloroform extraction and ethanol precipitation, and DNA integrity verified by agarose gel electrophoresis. Sex was available for 38 of the 68 animals, with 28 males and 10 females. Animal sizes ranged from 2.5 meters to 13.5 meters in length, with an average of 6.25 meters.

### Isolation of whale shark microsatellites

An oligonucleotide hybridization strategy was used to isolate microsatellite-containing sequences from whale shark genomic DNA (a modification of [23]). Genomic DNA (5 µg) was digested with *Hae*III and *Rsa*I, blunt-ended with Klenow and T4 DNA polymerase, dephosphorylated with calf intestinal phosphatase and purified using QIAquick (Qiagen). The forSNX (5′-CTAAGGCCTTGCTAGCAGAAGC-3′) and revSNX (5′-pGCTTCTGCTAGCAAGGCCTTAGAAAA-3′) linkers were annealed, then ligated to the blunt-ended DNA using T4 DNA ligase at room temperature overnight. The linkered DNA was purified using QIAquick and hybridized with 30-mer CA and GA biotinylated oligonucleotides in 12× SSC, 0.1% SDS hybridization buffer at 65 degrees for 2 hours. Repeat-containing DNA fragments were isolated using streptavidin coated magnetic beads (MagneSphere, Promega) according to the manufacturer's protocol. The beads were washed once wash at room temperature in 2× SSC, 0.1% SDS and once at 50 degrees with 1× SSC, 0.1% SDS. Purified DNA was eluted in 10 mM Tris at 95 degrees, and amplified by PCR using the SNX primer. The amplified DNA was cloned using the TopoTA kit (Invitrogen), and individual clones were sequenced and analyzed for the presence of repeats (Table 1). All microsatellite sequences isolated during the course of this work have been submitted to GenBank under the accession numbers FJ357449–FJ357456.

**Table 1:** Characteristics of whale shark microsatellite loci.

Locus	Repeat	N_a_	H_o_	H_e_	F_IS_
Rtyp1	(TG)_2_TC(TG)_6_TC(TG)…(TG)_9_…(TG)_9_TC(TG)_7_	5	0.687	0.681	−0.005
Rtyp2	(TG)_3_TC(TG)_12_TC(TG)_2_TC(TG)_8_CG(TG)_2_	7	0.731	0.714	−0.109
Rtyp3	(TG)_14_…(TG)_5_…(TG)_4_	4	0.802	0.546	−0.423
Rtyp4	(CT)_3_…TTTTCTGT(CT)_14_GTCT	4	0.472	0.402	−0.180
Rtyp5	(CA)_20_	7	0.853	0.874	0.065
Rtyp6	(GA)_41_	34	0.571	1.000	0.474*
Rtyp7	(CA)_3_TG(CA)_3_TA(CA)_3_(CT)_4_CC(CA)_19_	8	0.738	0.826	0.029
Rtyp8	(CA)_3_TGT(GC)_4_(CA_)9_TACA	3	0.446	0.455	0.020

Na indicates the number of alleles at each locus, Ho and He indicate observed and expected heterozygosities. Only Rtyp6, indicated with a *, deviates from Hardy-Weinberg equilibrium.

### Microsatellite genotyping

Following extraction, DNA samples were quantified using a nanodrop spectrophotometer, and 30 ng DNA per locus was amplified by PCR using primers against unique sequences flanking each microsatellite. Amplification employed 6-Fam labeled forward primers, and reverse primers tailed with the sequence GTGTCTT to promote 3′ nontemplated nucleotide addition (PIG-tailing) [24] (Table 2). PCR reactions were performed in 10 µl volumes with the following mix: 50 mM KCl, 10 mM Tris-HCl, 200 µM each dNTP, 50 µM each primer, and 0.1 U AmpliTaq Gold DNA polymerase (Applied Biosystems Inc). Magnesium concentrations and amplification profiles were established independently for each primer pair (Table 2). All reactions incorporated the GeneScan 350 ROX internal size standard (Applied Biosystems Inc.), and were run for 35 cycles in a Mastercycler gradient thermal cycler (Eppendorf). PCR products were resolved on an ABI 3730 DNA analyzer, and data analyzed using GeneMapper software (Applied Biosystems Inc.). Alleles showing stutter peaks were called as suggested by Haberl & Tautz [25]. Each sample was subjected to at least two independent analyses for all loci. In all cases, allele calls were identical when blinded to previous data.

**Table 2:** Primer sequences and PCR parameters for whale shark microsatellite loci.

Locus	Primers	Mg	Cycle Parameters
Rtyp1	Rtyp1ForF, 5′-AGGGGAGTGAATCTGTGGAAGTC-3′	2.0	94° 20″, 62° 20″, 72° 20″, 35 cycles
	Rtyp1RevT, 5′-GTGTCTTCGCAGCAAACATCGTCTCAGTG-3′		
Rtyp2	Rtyp2ForF, 5′-TCTTCCACTGTGTTCAAGTGTGTT-3′	2.0	94° 20″, 58° 20″, 72° 20″, 35 cycles
	Rtyp2RevT, 5′-GTGTCTTATATTCCATAGCTGCACTGAGGTCC-3′		
Rtyp3	Rtyp3ForF, 5′-GTTCAAATAGTGACTGGATGGAGAATGC-3′	2.0	94° 20″, 62° 20″, 72° 20″, 35 cycles
	Rtyp3RevT, 5′-GTGTCTTGGATGCAACTAACATACACATGTAATATGG-3′		
Rtyp4	Rtyp4ForF, 5′-TGGCGATGGTCTAACTTACATGAGC-3′	2.5	94° 20″, 58° 20″, 72° 20″, 35 cycles
	Rtyp4RevT, 5′-GTGTCTTTCCGGACTTCATCACCCTAACATG-3′		
Rtyp5	Rtyp5ForF, 5′-TGACTTATGTCATCTGCATTTCAACC-3′	1.5	94° 20″, 56° 20″, 72° 20″, 35 cycles
	Rtyp5RevT, 5′-GTGTCTTCCTACCCTGATGCAATTTGTATG-3′		
Rtyp6	Rtyp6ForF, 5′-TTGAGGGAGTGCAGTGAAGGG-3′	1.5	94° 20″, 56° 20″, 72° 20″, 35 cycles
	Rtyp6RevT, 5′-GTGTCTTTGCATTCAACCTATCTGGTCCTG-3′		
Rtyp7	Rtyp7ForF, 5′-TGTACCTGTTGTATAGCATTGGAAGG-3′	1.5	94° 25″, 58° 25″, 72° 25″, 35 cycles
	Rtyp7RevT, 5′-GTGTCTTGGGATTTATAAATAGCCACATTGACTG-3′		
Rtyp8	Rtyp8ForF, 5′-CGATTGGTTAACTAAGTCAGAGTATGG-3′	1.5	94° 20″, 60° 20″, 72° 20″, 35 cycles
	Rtyp8RevT, 5′-GTGTCTTCGAAGTCTTTGCCCACTCACTTAAC-3′		

Mg indicates the magnesium concentration for amplification.

### Analysis of microsatellite loci

Although a total of 68 whale shark samples were analyzed, some samples did not yield sufficient quantities of DNA to analyze all loci, and some DNAs did not yield useful data for all loci. Different numbers of samples are therefore reported for the different loci. In all cases, where fewer than 68 animals were used for analysis, this is indicated in the methodology. In individual tests for genetic differentiation (Structure and PCA), the single South African animal was treated individually. In analyses where animals were grouped into populations, this animal was included with the Indian Ocean group. Input files for various software were constructed using the program Create when possible [26]. Locus data was initially checked for the presence of null alleles, stuttering and small allele dominance using the program MicroChecker 2.2.3 [27]. Locus statistics and concordance with Hardy-Weinberg Equilibrium were calculated using FSTAT [28]. GENEPOP 4.0 was used to test for linkage disequilibrium using the log likelihood ratio statistic (G-test), with the parameters, dememorization number = 10,000, number of batches = 1,000, number of iterations per batch = 10,000 [29].

### Tests for population differentiation and genetic distance

A Bayesian approach using genotype data for individual animals was performed to detect any population structure across the entire data set using the program STRUCTURE 2.2 [30]. STRUCTURE was run with assumptions of K = 1–5, using a burnin length of 50,000 and a run of 50,000 steps. All runs were repeated in triplicate at each K, and results were consistent across runs. Principle Components Analysis (PCA) is a method of detecting patterns of variation in complex data sets, and determining the extent to which individual patterns contribute to the variance of the data as a whole. PCA was conducted on individual multilocus genotypes using GenAlEx 6.1 with the standardized covariance method [31]. All individuals that were genotyped at six or more loci (N = 43) were included, and the analysis was run without the Rtyp6 locus (see Results).

Traditional tests for population differentiation were performed by calculation of F-statistics using FSTAT and Microsatellite Analyzer (MSA) 4.05 [32]. As numbers of animals from individual populations were small, animals were pooled into three same-ocean groups for analysis of population differentiation - Pacific, Caribbean and Indian. FSTAT was run without assuming Hardy-Weinberg equilibrium between populations, for 3,000 permutations. A matrix representing Nei's standard genetic distance (D_s_) was produced using the program POPULATIONS 1.2.30 [33], [34]. This analysis used animals scored for at least 4 loci (N = 59).

### Effective population size and probability of identity

Calculation of effective population size (N_e_) was performed using ARLEQUIN 2.0 [35]. Data from all individual whale sharks was pooled into a single data set to obtain a global estimate. The number of mutations per generation, Theta (*θ*_H_), was calculated from the expected homozygosity (Hom_E_). Assuming that the population is in mutation-drift equilibrium, Theta(θ_H_) is *θ*_H_ = (1−Hom_E_)/Hom_E_, where Hom_E_ = 1−H_E_, and H_E_ is expected heterozygosity. To evaluate the utility of microsatellite genotypes as individual genetic tags, we estimated the probability of identity for individual loci and over all eight loci using Cervus 3.0.3 [36], [37].

## Results

### Isolation of whale shark microsatellites

Whale sharks are the only genus within their family, Rhincodontidae, and microsatellites had not previously been described from this species. Microsatellites have been isolated from members of the sister families Stegostomatidae and Ginglymostomatidae, but microsatellite sequences are highly unlikely to be conserved across families [38]–[44]. We therefore chose to isolate whale shark microsatellites using a repeat oligonucleotide hybridization strategy (a modification of [23]). Sequencing of 98 individual selected clones yielded 77 unique sequences, of which 20 carried a repeat of 10 units or more. Most were complex repeats of mixed nucleotide composition. These 20 loci were tested for polymorphism within a preliminary panel of whale shark DNAs. Eight loci, Rtyp1 through Rtyp8, were found to be polymorphic and to give good amplification with a minimum of stutter peaks (Table 1 and Table 2). The number of alleles (N_a_) per locus ranged from 3 for Rtyp8, to 34 for Rtyp6, with a mean of 9. The Rtyp6 locus was unusual in its degree of polymorphism, and N_a_ excluding Rtyp6 was 3–8, with an average of 5.4. The range for observed heterozygosity (H_o_) was 0.45–0.85, and for expected heterozygosity (H_e_) 0.40–1.00. The loci were analyzed with the program MicroChecker, and with the exception of Rtyp6 all conformed to Hardy-Weinberg equilibrium, with no evidence for scoring error, null alleles or large allele dropout [27]. Rtyp6 displayed significant heterozygote deficiency (p<0.0001), suggesting the possibility of null alleles at this locus.

Analysis of each locus pair across all populations using the program GENEPOP found no evidence for linkage disequilibrium, indicating that all microsatellite loci are unlinked and segregating independently (data not shown). Allelic richness (R_S_), a measure of genetic diversity that describes the number of alleles per locus independent of sample size, could not be calculated individually for several populations due to small sample size. This value was therefore examined after the animals had been pooled into ocean-specific populations, as described below. Values for R_S_ ranged from 2.0 to 8.7 (Table 3). A small number of private alleles were identified, with the majority of these found at the highly polymorphic Rtyp6 locus (Table 4). For other loci, private alleles were few in number, and were distributed across loci and across populations. As null alleles were suspected at locus Rtyp6, all data analysis was performed both with and without this locus. For none of the analyses did the exclusion of Rtyp6 change the general trend of the data, or alter the overall conclusions. The data are reported with Rtyp6 included, unless stated otherwise. In this study, no two animals had identical genotypes, ruling out the possibility that any animal was sampled twice.

**Table 3:** Allelic richness.

Locus	Pacific	Caribbean	Indian	All
Rtyp1	4.041	3.000	3.572	3.606
Rtyp2	3.740	5.000	3.945	4.096
Rtyp3	3.190	3.667	3.002	3.083
Rtyp4	2.495	2.000	2.087	2.143
Rtyp5	5.141	4.000	5.192	5.047
Rtyp6	7.878	5.470	8.659	8.544
Rtyp7	4.961	4.000	5.213	5.341
Rtyp8	2.000	2.000	2.181	2.114

**Table 4:** Private alleles.

Pop	Locus	Allele	Freq
Pacific	Rtyp1	218	0.100
	Rtyp4	151	0.100
	Rtyp6	221	0.125
	Rtyp6	169	0.250
	Rtyp6	181	0.100
	Rtyp6	235	0.100
Caribbean	Rtyp2	218	0.200
	Rtyp6	281	0.200
Indian	Rtyp2	237	0.125
	Rtyp2	222	0.033
	Rtyp4	160	0.063
	Rtyp6	213	0.063
	Rtyp6	255	0.063
	Rtyp6	284	0.500
	Rtyp6	251	0.500
	Rtyp6	167	0.200
	Rtyp6	204	0.100
	Rtyp6	243	0.100
	Rtyp6	217	0.071
	Rtyp6	223	0.071
	Rtyp6	237	0.143
	Rtyp6	257	0.143
	Rtyp6	229	0.125
	Rtyp6	239	0.250
	Rtyp6	245	0.125
	Rtyp8	211	0.063

### Microsatellite frequency across shark species

It was reported previously that levels of genetic variation are generally low in sharks, in comparison to other fishes [45]. For example, microsatellite isolation in the sandbar shark required the screening of large numbers of microsatellites to find polymorphic sequences [46]. Despite this, other studies have identified long and highly polymorphic repeats from some shark species [47], [48]. In the present study, microsatellites were isolated from whale shark DNA with relative ease, and moderate levels of genetic variation were found. Approximately half of the microsatellites tested were polymorphic. Microsatellite isolation protocols have advanced technically, however, and current enrichment protocols make it difficult to compare with previous studies. Table 5 shows a comparison of microsatellite locus characteristics across multiple shark species [38]–[40], [42], [44], [48]–[53]. Overall, the whale shark microsatellites described here approximate those from most other shark species in repeat length and levels of heterozygosity, while the number of alleles is somewhat lower than the average.

**Table 5:** Microsatellite statistics for other shark species.

Species	N_MS_	Longest Repeat	Average Repeat	N_a_	Avg N_a_	H_o_	Avg H_o_	H_e_	Avg H_e_	Reference
Whale shark										
(*Rhincodon typus*)	8	41	17.2	3–34	9.0	0.44–0.85	0.66	0.40–1.00	0.69	This work
Spiny dogfish										
(*Squalus acanthias*)	6	12	9.7	3–9	5.8	0.37–0.84	0.59	0.51–0.81	0.68	[49]
Zebra shark										
(*Stegostoma fasciatum*)	9	32	20.1	3–22	9.6	0.40–0.97	0.63	0.34–0.92	0.71	[38]
Nurse shark										
(*Ginglymostoma cirratum*)	9	26	12.0	2–15	5.0	0.17–0.90	0.55	0.16–0.92	0.54	[39]
Sandtiger shark										
(*Carcharias taurus*)	5	20	13.4	3–9	6.2	0.29–0.75	0.62	0.28–0.73	0.61	[50]
White shark										
(*Carcharodon carcharias*)	5	23	18.2	2–10	5.4	0.45–0.95	0.70	0.51–0.83	0.66	[40]
Shortfin mako										
(*Isurus oxyrinchus*)	5	53	22.4	14–57	31.6	0.77–0.91	0.86	0.82–0.96	0.89	[42]
Blacktip shark										
(*Carcharhinus limbatus*)	8	NA	NA	4–42	14.1	0.10–0.96	0.50	0.09–0.96	0.50	[51]
Sandbar shark										
(*Carcharhinus plumbeus*)	5	42	22.8	4–39	22.6	0.63–1.00	0.87	0.57–0.96	0.85	[52]
Spot-tail shark										
(*Carcharhinus sorrah*)	5	28	19.2	4–24	9.8	0.12–0.82	0.50	0.16–0.95	0.54	[44]
Australian black-tip shark										
(*Carcharhinus tilstoni*)	5	19	12.0	5–24	10.8	0.44–0.78	0.65	0.54–0.92	0.73	[44]
Lemon shark										
(*Negaprion brevirostris)*	4	33	25.2	19–43	28.5	0.68–0.87	0.77	0.69–0.90	0.78	[48]
Bonnethead shark										
(*Sphyrna tiburo*)	4	NA	NA	6–35	13.5	0.51–0.87	0.65	0.55–0.96	0.69	[53]
Averages for all species		29.9	17.5		13.2		0.66		0.68	

Values are given only for dinucleotide repeats; only polymorphic loci are included. For complex repeats, the longest repeat was counted. NA indicates that the value was not given in the original reference.

### Individual-based tests for whale shark genetic structure

Initial tests for whale shark population structure used a Bayesian-clustering method to look for genetic structure using only genotypic data—considering each animal independently, regardless of the population grouping within which they were sampled. The program STRUCTURE analyzes allele frequencies across multiple loci, and assigns individuals to appropriate populations [30]. STRUCTURE was run multiple times, using the admixture model, under the hypothesis that the number of populations (K) contained within the whale shark data set was between 1 and 5. Values for ln Pr(X/K) varied little for the different estimates of K, indicating that individuals could not be partitioned into discrete genetic clusters. Rather, the STRUCTURE results suggest that whale sharks comprise a single genetic cluster.

**Figure 2:**
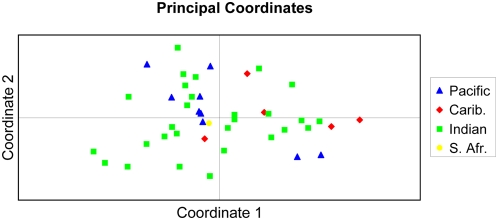
Principle Component Analysis (PCA) of individual whale shark multilocus genotypes performed using GenAlEx 6.1. Animals were analyzed as individuals, but are color-coded here by ocean populations for ease of interpretation. Pacific samples were from the eastern Pacific (Galapagos, Cocos islands and La Paz), Caribbean samples (Carib) were from Utila, Indian samples were from Veraval, Seychelles, Ningaloo, Djibouti and Maldives, and the single South Africa sample (S. Afr.) is coded independently.

The whale shark microsatellite data was subjected to Principal Components Analysis using the program GenAlEx 6.1, which found that the first two axes explained 24.7% and 21.6% of the total variance, respectively, representing nearly half of the of the genetic variance of the whole data set. However, the PCA analysis found no marked clustering of individuals, by sampling location or other factors (Figure 2). Whale sharks sampled from the Indian Ocean, including individuals from Veraval, Seychelles, Ningaloo, Djibouti and Maldives, spanned the PCA plot and broadly overlapped individuals sampled from the Caribbean (Utila) and eastern Pacific (Galapagos, Cocos Island, and La Paz). The single individual from South Africa was centrally located in the canonical plot. These data indicate that there is no discernable genetic difference within these samples between animals found at the various sites. Annotating the PCA plot specifically for those animals for which sex is known, showed no distinct segregation of males and females (data not shown).

### Population-based tests of genetic differentiation

In this study, small sample numbers precluded analyzing individual sampling locations as populations of whale sharks. Since the Bayesian analysis programs did not discern inherent structure among the individual whale sharks in our data set, the animals were grouped for further analysis into three ocean-specific populations—Pacific (P, N = 11), consisting of samples from La Paz, Galapagos and Cocos Island; Caribbean (C, N = 7), consisting of samples from Utila and Florida; and Indian (I, N = 50), consisting of samples from Djibouti, Seychelles, Maldives, Veraval and Ningaloo (Figure 1). The single South African shark was included in the Indian population, based on satellite tagging studies that showed these animals move north along the coast of East Africa [54]. Analysis of the pooled populations was performed using MSA and FSTAT, and the resulting F_ST_ values are presented in Table 6. F_ST_ values between the Pacific and Indian Ocean populations were quite low, with P×I = −0.0022, while Caribbean animals showed somewhat higher F_ST_ values against both Pacific and Indian Ocean populations (P×C = 0.0387 and C×I = 0.0296). F_ST_ values for P×I and P×C were not statistically significant, while the value for C×I approached statistical significance (p = 0.0495). The Caribbean animals may therefore be more differentiated from Indian Ocean sharks than they are from Pacific, or than Pacific and Indian are from each other. Those animals for which sex is known were analyzed separately as male and female populations using FSTAT; this analysis did not detect any statistically significant genetic difference between males and females (data not shown).

**Table 6:** FST matrix for whale shark populations.

	Pacific	Caribbean	Indian
Pacific	0	0.0387	−0.0022
Caribbean	0.0569	0	0.0296
Indian	0.5028	0.0495	0

Numbers above the diagonal are pairwise FST values; numbers below the diagonal are p values for each pairwise comparison.

Nei's standard genetic distance (D_S_), which describes the similarity between two groups of individuals based on allele frequencies using the Infinite Alleles Model, was calculated using the program POPULATIONS 1.2.30. Pairwise values for D_S_ were P×C = 0.248, C×I = 0.169 and P×I = 0.078. These data indicate a lack of strong genetic differentiation between any of the analyzed whale shark populations.

### Effective population size

The effective population size (N_e_) reflects the number of breeding adults and the potential for inbreeding and genetic drift in the species. Effective population size was estimated based on the *θ*_H_ value, which is *θ*_H_ = 4N_e_μ, where μ is the mutation rate. We used a mutation rate of 1×10^−3^ mutants/generation/locus [55]–[58], which generates an effective population estimate of 103,572 with a standard error range of 27,401–179,794 animals.

### Probability of identity

Microsatellite analysis can provide sufficient genetic resolution to identify individual animals within a population. Such “genetic tagging” can aid in identifying previously censused individuals and estimating population sizes when other methods of identification or tagging are difficult [59]–[61]. Establishment of a genetic database for whale sharks is a long-term goal that requires the ability to identify individual animals resampled over time. The program CERVUS 3.03 was used to calculate the probability of identity, the likelihood that each whale shark carries a unique pattern of genetic markers across the loci described here. For individual loci, the probability of identity ranged from 0.41 (for Rtyp4) to 0.0041 (for Rtyp6), and the combined probability of identity for the eight loci was 7.64×10^−9^. This value indicates that the possibility that any two samples showing the same genetic pattern across all loci do not come from the same animal is nearly 1 in 1 billion. As this value undoubtedly exceeds the whale shark population size by several orders of magnitude, these loci provide unique genetic tags for individual whale sharks.

## Discussion

### Genetic population structure

Microsatellite analysis of whale sharks sampled primarily at feeding aggregations around the world showed little genetic differentiation in this study. The Pacific and Indian Ocean populations were very similar based on F_ST_ value (P×I = −0.0022), while F_ST_ values for the Caribbean population were somewhat larger when compared to both the Pacific and Indian animals (C×P = 0.0387; C×I = 0.0296). Only the C×I population approached statistical significance for genetic differentiation (p = 0.0495). It is likely this value would be more highly significant if additional Caribbean animals were available for analysis. F_ST_ for C×I remains low, however, indicating subtle differentiation between Caribbean and Indian whale sharks. Despite this finding, the data show that there has been significant gene flow between geographically disparate populations. The individual-based analyses (STRUCTURE and PCA) also indicate no clear genetic clusters of whale sharks based on sampling location (Figure 2). Gene flow between geographic sampling populations could be mediated directly, by individual animals traversing large distances to interbreed with distant populations, or could be more incremental, as animals breed with near neighbor populations and their offspring subsequently move to yet more distant areas. As data become available about additional whale shark aggregation sites, it appears that a band of whale sharks spanning the mid-latitudes is plausible.

High rates of gene flow are typical in large, vagile marine species, which have few barriers to migration between populations. For some species, this migratory activity supports a single panmictic population that shows little genetic variability. For example, harbour porpoises (*Phocoena phocoena*) were found to consist of a single continuous population displaying isolation by distance throughout the North Atlantic, with genetically distinct Iberian and Black Sea populations only [62]. For other species, strong evidence for population differentiation has been found by genetic analysis, despite apparent high rates of migration. Analysis of swordfish (*Xiphias gladius*) mitochondrial DNA showed that fish from the Pacific, Mediterranean, Northern Atlantic and Southern Atlantic comprise four genetically distinct populations [63]. Bryde's whales (*Balaenoptera brydei*) have a lifestyle seemingly similar to that of whale sharks, as they inhabit tropical and warm temperate waters, and have been shown to undertake equatorial migrations. Nonetheless, microsatellites and mitochondrial DNA analysis was used to show that these whales comprise distinct oceanic populations in the Northern Pacific, Southwestern Pacific, Southeastern Pacific and Indian oceans, with low levels of gene flow [64]. Analysis of mitochondrial and nuclear DNA sequences from bluefin tuna (*Thunnus thynnus*) has yielded conflicting results for population structure between the Mediterranean and northwest Atlantic [65], [66]. Most recently, microsatellite analysis of larval fish from these two regions demonstrated significant spawning site fidelity, despite population intermixing on feeding grounds [67]. Among shark species, lemon sharks studied at four sites in the Western Atlantic and Caribbean, showed little evidence of population structure. Though F_ST_ values were statistically significant between three of the four populations, the values were quite small, ranging from 0.005 to 0.034 [48]. Mitochondrial and nuclear DNA analysis in the scalloped hammerhead (*Sphyrna lewini*) showed significant genetic divergence between Atlantic and Indo-Pacific populations, and identified a cryptic species within the northwest Atlantic [68].

### Evidence for female site fidelity

Two previous studies have employed mitochondrial DNA sequence analysis to compare whale shark populations. Analysis of the mitochondrial control region was used to study the relatedness of 41 whale sharks in the Gulf of California [69]. This study found high levels of genetic variation in the sharks analyzed (females and juveniles), but saw no evidence for population structure in the region. Castro et al sequenced the mitochondrial control region from a population of 70 whale sharks, a study similar in sample number and geographical distribution to that presented here [70]. This analysis found high haplotype and nucleotide diversity (44 haplotypes), with the most common haplotype distributed globally. Little population structure was evident between the Indian and Pacific oceans, but the study found statistically significant differences in haplotype frequency between the Atlantic and Indo-Pacific populations (Φ_ST_ = 0.107, p<0.001). These data indicate significant gene flow between Indian and Pacific Ocean populations, with reduced levels of interaction with Atlantic animals, a result that parallels the data from our microsatellite analysis. It is possible that the somewhat greater degree of Caribbean/Atlantic population differentiation seen using mitochondrial DNA markers, in comparison to the microsatellites described here, indicates some level of female site fidelity. This hypothesis must be qualified, however, given the small number of animals analyzed in both studies.

Female philopatry is found in numerous shark species. White sharks are highly migratory, for example, yet genetic analysis has shown strong population structure. Mitochondrial DNA analysis of sharks from South Africa found evidence for population differentiation in comparison to sharks from Australia/New Zealand, with F_ST_ values of 0.81 between South Africa and Australia, and of 0.89 between South Africa and New Zealand [40]. Strikingly, microsatellite analysis of these same populations revealed no significant genetic differences between populations. These data suggest male-biased gene flow, while indicating that female white sharks are highly philopatric. In blacktip sharks, microsatellites and mitochondrial DNA were used to examine the genetic makeup of juvenile sharks from nurseries located throughout the Gulf of Mexico and Western Atlantic [51]. Significant differentiation (F_ST_ = 0.063–0.067) of nuclear markers was found, particularly between Belize and the other nurseries. Genetic differentiation was far greater using analysis of mitochondrial DNA, however, providing evidence for female philopatry. Any demonstration of female philopatry in whale sharks must await the sampling and analysis of larger numbers of these animals.

### Ecological population structure

Satellite tracking studies of whale sharks have demonstrated both short and long range migratory movements, which support the gene flow inferred with microsatellite markers. Sharks tagged near Taiwan, at Gladden Spit off the coast of Belize, and at Ningaloo reef in Western Australia, all recorded short range movements within their ocean basins [11], [12], [14], [15], [54]. Animals tagged at Ningaloo, for example, traveled northeast towards Indonesia, while Belize animals moved to other regions of Central America and to the Yucatan [12], [14]. Sharks tagged off the coast of KwaZulu-Natal, South Africa moved several hundred miles up the eastern coast of Africa to Mozambique [54]. Longer migrations were recorded in the Seychelles, where one tagged individual moved over 3000 km into the Indian Ocean [13]. Most strikingly, Eckert and Stewart tagged 17 sharks in Gulf of California, of which four moved into the western Pacific where they covered several thousand kilometers, and one animal traveled nearly 13,000 km into the North Pacific [10]. These data most strongly support genetic homogeneity based on segmental gene flow, punctuated by occasional long-distance migrations. The sexes of the tagged animals were not reported in all of the above studies, and therefore the numbers remain small to draw conclusions, yet no unique patterns appear to distinguish the migratory habits of male versus female whale sharks.

Ecological data also support frequent movements of whale sharks between populations. Our sample set displays a striking absence of females, with 2.8 males for every 1 female, reflecting the observation that most feeding aggregations are composed largely of immature males. For example, most sharks observed at Ningaloo Reef, at Gladden Spit, and off the coast of Djibouti are juvenile males [9], [14], [19], [20], [71], [72]. Large concentrations of adult female sharks have to date been found in only two locations, the southern end of the Gulf of California near Bahia de La Paz, and the Galapagos Islands [7](M. Levine, personal communication). The majority of females in our sample set originated from these two locations, with a few smaller females found in Djibouti. Animals from Ningaloo and from the Maldives contained no females at all, while the other groups were largely unsexed. Whale sharks therefore segregate by size and sex, and since the most well-studied aggregations represent nonfunctional populations, breeding must occur outside these aggregations.

### Effective population size

While early estimates proposed that the world population of whale sharks might number no more than a few thousand, identification of larger aggregations of animals in previously unknown locations has revised that estimate upwards. Data from tagging and mark-recapture studies has shown that surprisingly few animals are resighted at most locations. Of 72 whale sharks tagged at Gladden Spit, only 17 individuals were resighted over 5 years [14]. We have used microsatellite data to estimate the effective population size at 27,401–179,794 animals. Despite the different methodology employed, this estimate agrees relatively well with that calculated from mitochondrial DNA analysis of 119,000–238,000 females, or 238,000–476,000 total animals [70]. Both results must be viewed with caution, however, given the small number of samples used, and the wide error rate implicit in such calculations. In particular, the lack of a definitive mutation rate for microsatellites in shark species indicates that this estimate be considered a rough approximation at best. We have shown that the 8 microsatellite loci described here can accurately be used for genetic tagging of whale sharks with a probability of identity of 7.64×10^−9^. As has been demonstrated in other sharks, seals and rays, the ability to genetically identify any individual within a population can discern breeding structure, paternity and sibship [59], [73]–[76]. Compiling such information across investigators and across geographic populations of animals, as has been done for photo identification [16], [77], would allow fine-scale genetic analysis currently impossible with the sample sets available.

### Implications for conservation management

The work presented here describes the first identification of microsatellite loci in the whale shark, and the use of these loci to analyze population structure across a panel of whale shark DNAs from three different ocean basins. This much-needed first look at whale shark population structure using nuclear markers showed little genetic differentiation between geographic populations. Rather, the data confirm a history of gene flow between populations, supporting migration and interbreeding between these seemingly disparate groups. Such data are supported by satellite tracking studies that show frequent mid-range and periodic long-range migrations. Though this level of gene flow is sufficient to genetically normalize populations, it is unlikely to be sufficient to reestablish depleted populations. As whale sharks cross geographic and political boundaries in their movements, international protection should be sought to ensure the continued survival of this species. In addition, it should be kept in mind that genetic methods of population study reflect only the history of the species. They cannot detect more recent changes in behavior that may be caused by overfishing, habitat disruption, tourism, or other anthropogenic activities currently impacting whale shark populations.
